# Spread of Influenza Virus A (H5N1) Clade 2.3.2.1 to Bulgaria in Common Buzzards

**DOI:** 10.3201/eid1810.120357

**Published:** 2012-10

**Authors:** Atanaska Marinova-Petkova, Georgi Georgiev, Patrick Seiler, Daniel Darnell, John Franks, Scott Krauss, Richard J. Webby, Robert G. Webster

**Affiliations:** Regional Diagnostic Laboratory on Avian Influenza and Newcastle Disease in Birds, Varna, Bulgaria (A. Marinova-Petkova);; National Diagnostic and Research Veterinary Medical Institute, Sofia, Bulgaria (A. Marinova-Petkova, G. Georgiev);; and St. Jude Children’s Research Hospital, Memphis, TN, USA (P. Seiler, D. Darnell, J. Franks, S. Krauss. R.J. Webby, R.G. Webster)

**Keywords:** Highly pathogenic avian influenza, HPAI, H5N1, clade 2.3.2.1, Europe, common buzzard, influenza, Buteo buteo, Bulgaria, viruses, birds, avian

## Abstract

On March 15, 2010, a highly pathogenic avian influenza virus was isolated from the carcass of a common buzzard (*Buteo buteo*) in Bulgaria. Phylogenetic analyses of the virus showed a close genetic relationship with influenza virus A (H5N1) clade 2.3.2.1 viruses isolated from wild birds in the Tyva Republic and Mongolia during 2009–2010. Designated A/common buzzard/Bulgaria/38WB/2010, this strain was highly pathogenic in chickens but had low pathogenicity in mice and ferrets and no molecular markers of increased pathogenicity in mammals. The establishment of clade 2.3.2.1 highly pathogenic avian influenza viruses of the H5N1 subtype in wild birds in Europe would increase the likelihood of health threats to humans and poultry in the region.

## Abstract 2

Detection of this highly pathogenic clade in Europe poses a health threat to poultry and humans

Wild aquatic birds are considered natural reservoirs of all known influenza virus subtypes (*1*). Highly pathogenic avian influenza viruses (HPAIVs) usually cause asymptomatic infections in waterfowl. Compared with that for poultry, the number of reported outbreaks of HPAIVs in wild birds (aquatic or terrestrial) before 2002 was low; 1 influenza A (H5N3) outbreak occurred in wild common terns (*Sterna hirundo*) in South Africa in 1961 (*2*), and H7 subtype HPAIV was isolated from a Saker falcon (*Falco cherrug*) in Italy in 2000 (*3*). In December 2002, a die-off of aquatic waterfowl caused by an HPAIV (H5N1) occurred in Penfold Park in Hong Kong. That event was followed by a second outbreak a week later in Kowloon Park, also in Hong Kong (*4*). Since then, cases of HPAIVs (H5 and H7 subtypes) in wild birds have been reported often.

In May 2005, a massive HPAIV (H5N1) outbreak occurred in wild aquatic birds in Qinghai Lake, western People’s Republic of China; 6,184 gulls, geese, great cormorants, and ruddy shelducks died (*5*). The lake is a staging area for migratory waterfowl, and the scientific community’s fear that the virus would spread during migration (*6*) was realized when the so-called Qinghai-like influenza virus A (H5N1) clade 2.2 spread to western Siberia in Russia and then to many countries in Asia, the Middle East, Europe, and Africa at the end of 2005 and during 2006, killing poultry flocks and wild birds.

The 2008 classification system used to describe the evolution and diversification of the HPAIVs (H5N1) that emerged from the A/goose/Guangdong/96 lineage (*7*) was updated in 2011. Phylogenetic analysis of all isolated influenza (H5N1) viruses showed that some of the 10 first-order clades (0–9) had stopped circulating in 2008 or earlier (clades 0, 3, 4, 5, 6, 8, 9), as had some second- and third-order groups of clade 2. Meanwhile, clades 1, 2.1.3, 2.2, 2.2.1, 2.3.2, 2.3.4, and 7 continued to evolve rapidly (*8*). Clade 2.3.2 is widely distributed in Asia, particularly in China, Hong Kong, Korea, Vietnam, Laos, Bangladesh, Nepal, Mongolia, and the Tyva Republic; it is also distributed in eastern Europe, mainly in Romania and Bulgaria (*8*,*9*). Tyva is part of the Siberian Federal District of Russia, which is located north of Mongolia. The remaining circulating influenza (H5N1) clades have specific geographic locations: clade 1 circulates in southern Vietnam and Cambodia; clade 2.1.3 in Indonesia; clade 2.2 in India and Bangladesh; clade 2.2.1 in Egypt; clade 2.3.4 in China, Hong Kong, Vietnam, Thailand, and Laos; and clade 7 in China and Vietnam.

Before 2006, no avian influenza outbreaks in poultry had been reported in Bulgaria; 4 cases of HPAIV (H5N1) clade 2.2 were confirmed in dead swans found in 4 regions of the country early that year (*10*). On March 15, 2010, the carcass of a common buzzard (*Buteo buteo*) containing HPAIV (H5N1) was found at St. Konstantin and Helena Black Sea Resort in Bulgaria and submitted to the Regional Diagnostic Laboratory on Avian Influenza (Varna, Bulgaria). The virus was characterized as clade 2.3.2.1.

## Materials and Methods

### Virus Isolation and Initial Characterization

Pooled lung, trachea, liver, cecal tonsil, and gizzard tissue from a common buzzard were injected into embryonated chicken eggs, and an avian influenza virus (A/common buzzard/Bulgaria/38WB/2010) was isolated. The isolate was subjected to hemagglutination inhibition (HI) assays, as specified in the World Organisation for Animal Health (OIE) Manual of Diagnostic Tests and Vaccines for Terrestrial Animals 2011 (*11*). The 50% egg infectious dose (EID_50_) was assayed in 10-day-old embryonated chicken eggs after a 40-hour incubation at 35°C and calculated according to Reed and Muench (*12*).

### Sequence Analysis

Viral RNA was extracted from virus-containing allantoic fluid by using the MagMax-96 AI/ND RNA extraction kit (Applied Biosystems/Ambion, Austin, TX, USA). Reverse transcription PCR was performed by using a universal set of primers (*13*,*14*); whole-genome sequencing was performed by using an Illumina Genome Analyzer (Illumina, San Diego, CA, USA), as described by Ducatez et al. (*15*).

### Phylogenetic Analysis

We performed a phylogenetic analysis of all gene segments of A/common buzzard/Bulgaria/38WB/2010. Sequences were retrieved from the National Center for Biotechnology Information influenza virus database (*16*) and aligned by using the ClustalW tool in MEGA4 (*17*). Phylogenetic relationships were estimated by using the MrBayes program (*18*) to apply a Bayesian method. The model of nucleotide substitution that best fits our data was selected by using the Modeltest 3.7 program (*19*).

### Antigenic Characterization and Pathogenicity Studies

We assessed the antigenic relationship of A/common buzzard/Bulgaria/38WB/2010 with other influenza (H5N1) viruses by performing HI assays with a panel of postinfection ferret antiserum against viruses from clades 2.3.2, 2.3.2.1, and 2.3.4; the panel was produced at St. Jude Children’s Research Hospital (Memphis, TN, USA; Table). All animal studies were conducted in United States Department of Agriculture–approved biosafety level 3 enhanced facilities at St. Jude Children’s Research Hospital.

**Table:** Hemagglutination inhibition titers used to compare the antigenicity of influenza A(H5N1) virus strain A/common buzzard/Bulgaria/38WB/2010 with other common strains*

Strain	Strain (clade)			
	A/common magpie/Hong Kong/ 5052/2007 (2.3.2.1)	A/Muscovy duck/Vietnam/ 1455/2006 (2.3.2)	A/duck/Laos/ 3295/2006 (2.3.4)	A/Japanese white-eye/Hong Kong/1038/2006 (2.3.4)
A/common magpie/Hong Kong/5052/2007	160	160	<40	<40
A/duck/Laos/3295/2006	<40	<40	160	80
A/Japanese white-eye/Hong Kong/1038/2006	<40	40	160	320
A/Muscovy duck/Vietnam/1455/2006	80	80	<40	<40
A/common buzzard/Bulgaria/38WB/2010	80	80	<40	<40

*Values represent titers that are the reciprocal of the lowest dilution of ferret antisera that inhibited hemagglutination caused by 4 hemagglutination units of the virus.

### Pathogenicity Tests in Chickens

The intravenous pathogenicity index test was conducted according to the OIE Manual of Diagnostic Tests and Vaccines for Terrestrial Animals 2011 (*11*) to determine whether A/common buzzard/Bulgaria/38WB/2010 is pathogenic in chickens. A natural route of infection study was performed in five 8-week-old specific pathogen–free chickens. A/common buzzard/Bulgaria/38WB/2010 (10^6^ EID_50_ in 0.5 mL) was administered to each bird as follows: 0.1 mL in the nares, 0.1 mL in the trachea, 0.2 mL in the throat, and 1 drop in each eye. The chickens were examined daily for clinical signs of disease.

### Pathogenicity Tests in Mice and Ferrets

The 50% mouse lethal dose was determined to assess the pathogenicity of the buzzard influenza (H5N1) virus in mammals. The experiment was conducted as described by Boon et al. (*20*), and the titer was calculated according to Reed and Muench (*12*).

We also performed a pathogenicity and transmissibility study in a ferret model (*21*). We collected nasal washes from all ferrets (i.e., inoculated ferrets, naive direct-contact ferrets, and naive respiratory droplet–contact ferrets) every other day and nasal swabs from only the donors (i.e., inoculated ferrets) on day 4 postinfection. EID_50_ was calculated according to Reed and Muench (*12*).

## Results

### Gross Pathologic Findings

The fresh common buzzard carcass that was initially found was in good condition, without any discharge or other clinical signs of disease. Necropsy revealed a sufficient quantity of yellow subcutaneous and abdominal fat tissue. Gross pathologic changes were not observed in the lungs, trachea, heart, liver, spleen, gizzard, stomach, pancreas, or intestines.

### Antigenic Characterization and Pathogenicity in Chickens

In the HI test, A/common buzzard/Bulgaria/38WB/2010 had a titer of 80 to antiserum from influenza (H5N1) clades 2.3.2 and 2.3.2.1 (Table). The intravenous pathogenicity index of A/common buzzard/Bulgaria/38WB/2010 was scored 3.0 because all 10 birds were found dead within 24 hours after inoculation. The natural route of infection study resulted in 2 chickens being found dead on day 2 postinfection, and the other 3 were sick. On day 3 postinfection, the remaining 3 chickens were found dead. The clinical signs observed before death were cloudy eyes, cyanosis of the exposed skin and wattles, edema of the face, and diarrhea.

### Pathogenicity in Mice and Ferrets

The 50% mouse lethal dose of A/common buzzard/Bulgaria/38WB/2010 was 30 EID_50_ (EID_50_ of the virus was 10^9^/mL). All 3 donor ferrets were shedding virus by day 5 postinfection; however, only 1 was still shedding virus by day 7 postinfection (Figure). Virus titers were not detected in the nasal washes of contact ferrets, indicating that A/common buzzard/Bulgaria/38WB/2010 is not transmissible by direct contact or respiratory droplets. All ferrets used in this study were healthy during the experiment and showed no clinical signs of disease; they were alert, playful, and eating and drinking normally.

**Figure:**
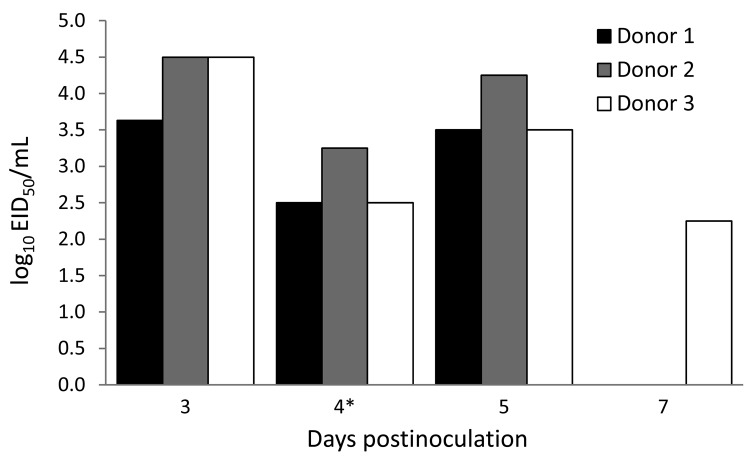
Virus titers in nasal washes or nasal swabs (*) of individual donor ferrets inoculated with A/common buzzard/38WB/2010. EID_50_, 50% egg infectious dose.

### Molecular Characterization of A/common buzzard/Bulgaria/38WB/2010

Genomic mutations identified in A/common buzzard/Bulgaria/38WB/2010 are listed in the Technical Appendix Table. The genome sequences of A/common buzzard/Bulgaria/38WB/2010 (H5N1) have been deposited in GenBank (accession nos. CY110851–CY110858).

#### Hemagglutinin

The virus’s hemagglutinin (HA) cleavage site had the polybasic amino acid sequence PQRERRRKRGLF, which is characteristic of HPAIVs (*22*). The cleavage site also had a K329 deletion (H5 numbering used throughout). However, none of the antigenic sites in HA1 of the H5 HA were mutated in comparison with A/whooper swan/Mongolia/2/2009, A/great crested grebe/Qinghai/1/2009, A/grebe/Tyva/3/2009, A/bar-headed goose/Qinghai/1/2010, and other closely related strains from clade 2.3.2.1 available in GenBank (site 1: amino acids 136–141; site 2: 152–153; site 3: 124–129, H5 numbering).

The receptor-binding pocket of HA1 retained residues E186, Q222, and G224, which preferentially bind to avian α-2,3-NeuAcGal receptors (*23*). All conserved residues within the HA receptor–binding domains of A/common buzzard/Bulgaria/38WB/2010 were identical to those of other clade 2.3.2 HA sequences available in GenBank (Technical Appendix Figure).

#### Neuraminidase

Compared with that of A/goose/Guangdong/1/96, the amino acid sequence of A/common buzzard/Bulgaria/38WB/2010s neuraminidase had a 20-residue deletion in the stalk region (residues 49–68), which was thought to be required for influenza viruses to adapt from wild aquatic birds to domestic chickens (*24*). This deletion causes a loss of the N terminal NQS glycosylation site (positions 50–52). Residues E119, H275, or N295 (N1 numbering) were not mutated, which suggests sensitivity to oseltamivir and zanamivir (*25*,*26*).

#### Polybasic Proteins

The A/common buzzard/Bulgaria/38WB/2010s *PB1* gene sequence contained 3 synonymous mutations, G204A, A750G, and G2052A; G204A in PB1’s open reading frame (ORF) caused a unique R37Q mutation in the polybasic (PB) 1-F2 protein. The PB1-F2 protein generated by the ORF plus 1 of the virus’s *PB1* genes is a 90-aa polypeptide. According to Zell et al. (*27*), PB1-F2 proteins consisting of >78 aa are intact and functional. The PB1-F2 N66S mutation, which is characteristic of increased viral pathogenicity and contributed to the high lethality of the 1918 pandemic influenza virus (*28*), was not observed in A/common buzzard/Bulgaria/38WB/2010. However, several PB2 mutations were present (Technical Appendix Table).

#### Nonstructural Proteins

The nonstructural (NS) 1 protein of A/common buzzard/Bulgaria/38WB/2010 is encoded by 230 aa. This sequence differs from other closely related NS sequences in the National Center for Biotechnology Information database in that it lacks the 5-aa deletion (residues 80–84) that became common in HPAIV (H5N1) NS sequences after they were first observed during 2001 in poultry in Hong Kong.

#### Matrix Proteins

The *M* gene–encoded ORF of matrix (M) 1 protein consists of 252 aa, and that of M2 consists of 97 aa. Residues 26L, 27V, 30A, 31S, and 34G of M2’s transmembrane region indicate that A/common buzzard/Bulgaria/38WB/2010 is an amantadine-sensitive strain (*29*).

#### Nucleoprotein

This virus’s nucleoprotein (NP) contained 4 aa substitutions, V33I, R293K, N395T and S450N. Normally, equine and avian influenza NPs have V33 (*30*). I33 is characteristic of human and swine influenza NPs (*30*,*31*), and V33I is a common amino acid substitution found in the pandemic viruses from 1918, 1957, 1968, 1977, and 2009 (*32*). K293 is also unique to human viruses (*32*,*33*). The NP of A/common buzzard/Bulgaria/38WB/2010 and that of the closely related A/whooper swan/Mongolia/1/2010 virus contain N450, a substitution found in North American bird strains; however, Eurasian strains typically contain S450.

### Phylogenetic Analysis

To determine the place of A/common buzzard/Bulgaria/38WB/2010 in the modern classification of influenza (H5N1) viruses, we phylogenetically analyzed HA nucleotide sequences of viruses representing all clades that had been reported as of the end of 2010. The HA of the Bulgarian isolate clustered with subtypes from clade 2.3.2.1 that were isolated in Mongolia and Tyva in 2009 and 2010 and originated from the A/black headed gull/Tyva/115/2009 and A/great crested grebe/Qinghai/1/2009 strains (Technical Appendix Figure).

To assess possible reassortment in the A/common buzzard/Bulgaria/38WB/2010 genome, we performed phylogenetic analysis of the remaining genes using the same group of viruses that had been used to make the HA tree. In the N1, PB1, PB2, PA, NS, and NP phylogenetic trees, A/common buzzard/Bulgaria/38WB/2010 clustered with the other subtype H5N1 viruses from clade 2.3.2.1 (data not shown). In the *M* gene tree, the Bulgarian subtype H5N1 virus clustered with the clade 2.3.4 subtypes from Guangxi, Hunan, Fujian, Shantou, and Hong Kong that were isolated in 2005, 2006, and 2008; all other subtype H5N1 viruses from clade 2.3.2.1 clustered in a separate group (Technical Appendix Figure). The evolutionary distance between the 2 groups of isolates in the M tree is long, indicating that the *M* gene of A/common buzzard/Bulgaria/38WB/2010 originates from a non–clade 2.3.2 ancestor.

## Discussion

The day after we received the HPAIV (H5N1)–containing common buzzard carcass, officials in Romania notified the OIE of an outbreak of an HPAIV (H5N1) in Letea Village (Danube Delta); 47 backyard chickens were found dead. These reports are considered to be the introduction of HPAIV (H5N1) clade 2.3.2.1 into Europe. Furthermore, the European Reference Laboratory on Avian Influenza and Newcastle Disease (Weybridge, UK) found that the HA gene sequence of the Bulgarian isolate is 99.9% similar to that of the Romanian isolates from Letea Village (*34*), confirming that both viruses are derived from a common source, which is most likely wild birds.

St. Konstantin and Helena Black Sea Resort, where the common buzzard carcass was found, is located on the border of Batova (43°21′11′′N, 27°57′33′′E), which is a complex of habitats typical for woodland bird species, waterfowl, and poultry. This area is defined as a bottleneck migration site of global importance because 3 flows of migratory birds meet over the Batova River Valley (*35*). Each spring (March–April), migratory waterfowl from Africa, Bosphorus, and the Dardanelle Straits stop at the lakes along the coast of the Black Sea in Bulgaria on their way north (*36*). Their next resting spot is the Danube Delta, where the Romanian outbreak occurred and <250 km from where the Bulgarian buzzard carcass was found. Thus, if we are correct in our hypothesis that birds from Via Pontica are the hosts that carried the HPAIV (H5N1) to Bulgaria and Romania, the virus most likely came from Tyva and Mongolia, where its ancestral viruses had been isolated, to Via Pontica by using >2 other overlapping flyways. Although this is the most likely scenario, it is still unconfirmed. To confirm this hypothesis, the pathogenicity of A/common buzzard/Bulgaria/38WB/2010 in ducks and its possible transmission among them must be defined, which will require additional biologic studies.

To trace the possible routes of introduction of HPAIVs (H5N1) into Bulgaria, the veterinary authorities and ornithologists from the Bulgarian Society for the Protection of Birds organized continuous monitoring of the bird areas along the Black Sea coast, near the Danube River and around the Ogosta Dam and Lom River. From January 1, 2010, through April 30, 2010, a total of 812 cloacal, fecal, and tissue samples from wild birds collected from these areas were tested for avian influenza virus; 269 samples were collected after March 15, 2010. All samples tested were negative for HPAIV (H5N1). Five carcasses of common buzzards found in different areas were submitted to the Regional Diagnostic Lab on Avian Influenza after March 15, 2010; only 1 was carrying HPAIV (H5N1).

Common buzzards are considered territorial birds that usually do not migrate long distances. A 3-year study conducted in southern England showed that local radio-tagged common buzzards forage within 1 km of their nests during their first winter; most of the birds that do not disperse make only brief excursions before they opt for a stay-at-home strategy, and most of those that disperse return to their natal area during the following breeding season (*37*). Long-term ornithologic studies conducted during 1979–2005 in Bulgaria, however, showed unambiguously that extensive autumn and spring migrations of common buzzards (as many as 42,100 birds) occur on the western Black Sea Via Pontica flyway (*35*,*38*). Kostadinova et al. reported ≈6 pairs of common buzzards breeding in the Batova habitat, but during autumn and spring migrations, ornithologists counted as many as 19,712 individuals of the species in the area (*35*). In most cases, common buzzards migrate singly or in loose flocks with other raptors, lesser spotted eagles, white pelicans, or black storks (*38*).

Migration of common buzzards in different parts of Europe appears to depend on the local climate; buzzards from northern Europe fly to the western Black Sea area during the winter season, whereas buzzards from Bulgaria fly south. Tracking bands belonging to common buzzards from Finland, Romania, and Israel have been found in Bulgaria (*39*). The buzzard infected with HPAIV (H5N1) in Bulgaria was not banded; however, even if it had been a migrant, the habitat nearest to St. Konstantin and Helena Black Sea Resort that provides different food sources is Batova, with an area of 38,132.8 ha (*35*). The migration of the common buzzards suggests that these birds are capable of spreading pathogens over long distances.

Our results show that chickens are highly susceptible to influenza virus A/common buzzard/Bulgaria/38WB/2010 (H5N1) and that the virus is highly pathogenic in them. Mammals appear not to be susceptible. Although buzzards can serve as intermediate hosts of HPAIV (H5N1) between migratory birds and poultry, the lack of gross pathologic findings in the buzzard carcass we examined indicates that the bird died shortly after infection. Thus, in this case, the buzzard could not have served as a reservoir of infection to spread the virus over a long distance. Additionally, the lack of poultry farms within 10 km of the area where the buzzard carcass was found may partially explain why no outbreak occurred. As part of a regular avian influenza surveillance plan, we tested 1,709 cloacal and fecal samples from mule ducks that were collected monthly during January 1, 2010–April 30, 2010, from 64 farms in 5 regions of Bulgaria (Plovdiv, Pazardjik, Stara Zagora, Haskovo, and Dobrich). No notifiable avian influenza viruses were isolated from any sample.

Since clade 2.3.2 was first isolated from a dead Chinese pond heron in Hong Kong in 2004, it has spread geographically and evolved genetically. A new fourth-order clade, 2.3.2.1, was recently identified, and A/common buzzard/Bulgaria/38WB/2010 was classified in this clade (*8*). The question that arises now that clade 2.3.2.1 has spread from Asia to Europe is whether it can cause a scenario similar to that caused by clade 2.2 from 2005–2006, when HPAIV (H5N1) killed millions of birds in Asia, Europe, and Africa. Although no new HPAIV (H5N1)–related events have been reported in Europe since March 2010, some of the aspects of the 2.3.2.1 clade make it difficult to predict the consequences of the clade’s arrival on the continent. For example, this clade is already widely distributed in Asia and is being perpetuated in many wild bird species, which is a prerequisite for long-distance distribution through migration. Wild bird species infected with HPAIV (H5N1) from clade 2.3.2.1 include gray herons, peregrine falcons, and great egrets in Hong Kong; whooper swans, ruby shelducks, and bar-headed geese in Mongolia; and grebes and black-headed gulls in Tyva (*8*).

The potential of clade 2.3.2.1 HPAIV (H5N1) to cause an outbreak is heightened because vaccines currently in use do not efficiently protect poultry flocks from a strain of this clade that was recently identified in Vietnam (*40*). Now that clade 2.3.2.1 has spread to Europe, implementing active surveillance plans in all high-risk areas and monitoring the wild birds in the region will play key roles in early detection of incidences of HPAIV (H5N1) infection and in prevention of outbreaks. The expansion of the geographic distribution of HPAIV (H5N1) in wild birds and poultry and the virus’s repeated interspecies transmission to humans make this virus a substantial pandemic threat.

Technical Appendix Amino acid substitutions in A/common buzzard/Bulgaria/WB38/2010 and comparison of deduced amino acid sequences of the hemagglutinin protein of A/common buzzard/Bulgaria/38WB/2010 and closely related sequences of 4 other viruses (A/great crested grebe/Qinghai/1/2009, A/grebe/Tyva/3/2009, A/whooper swan/Mongolia/8/2009, and A/whooper swan/Mongolia/2/2009).
