# Supplementary material for: Spread of Influenza Virus A (H5N1) Clade 2.3.2.1 to Bulgaria in Common Buzzards
Source: Emerg Infect Dis. 2012 Oct;18(10):1596–602. doi: 10.3201/eid1810.120357 (PMC3471626; doi:10.3201/eid1810.120357)
Supplement: Technical Appendix — Amino acid substitutions in A/common buzzard/Bulgaria/WB38/2010 and comparison of deduced amino acid sequences of the hemagglutinin protein of A/common buzzard/Bulgaria/38WB/2010 and closely related sequences of 4 other viruses (A/great crested grebe/Qinghai/1/2009, A/grebe/Tyva/3/2009, A/whooper swan/Mongolia/8/2009, and A/whooper swan/Mongolia/2/2009). [file 12-0357-Techapp-s1.pdf]

Article DOI: http://dx.doi.org/10.3201/eid1810.120357

Spread of Influenza Virus A (H5N1) Clade 2.3.2.1 to Bulgaria in Common Buzzards

Technical Appendix

Technical Appendix Table. Amino acid substitutions in A/common buzzard/Bulgaria/WB38/2010

Gene segment	Amino acid substitution	Comment
HA	T195I*	
NA	V321I*	
	D398N*	
PB1	N158S	Also in other viruses isolated in Tyva in 2009 and 2010.
	D464N*	
	S573T*	
PB1-F2	R37Q*	
PB2	V231I	Also in closely related viruses isolated in Tyva and Mongolia in 2009 and 2010 and in those from Hong Kong and Qinghai.
	T339M	Also in A/duck/Hunan/8/2008.
	Q628L*	
	K718R*	
PA	V44I*	
	T263A	Also in closely related viruses isolated in Tyva and Mongolia in 2009.
NS1	H17Y*	
	D207N*	
	D70	Also in closely related viruses isolated in Qinghai, Tyva, and Mongolia in 2009 and 2010.
	V112	
	M123	
	V128	
	S171	
NS2	L35	
NP	V33I	I33 is a characteristic of human viruses and swine viruses.
	R293K	K293 is a characteristic of human viruses.
	N395T	Also in closely related viruses isolated in Tyva and Mongolia.
	S450N	N450 is a characteristic of North American avian influenza strains.
M1	V115	Characteristic of avian viruses.
	T137	
M2	V28	Characteristic of human viruses (*1*).

*Amino acid substitutions that are not characteristic of other closely related viruses (i.e., unique mutations). HA, hemagglutinin; NA, neuraminidase; PB, polybasic; PA, protective antigen; NS, nonstructural; NP, nucleoprotien; M, matrix.

```
                                          10         20         30         40         50         60
                                  ....|....| ....|....| ....|....| ....|....| ....|....| ....|....|
A/Common Buzzard/BG/38WB/2010     DHICIGYHAN NSTEQVDTIM EKNVTVTHAQ DILEKTHNGK LCDLNGVKPL ILKDCSVAGW
A/great crested-grebe/Qinghai/    .......... .......... .......... .......... .......... ..........
A/grebe/Tyva/3/2009               .......... .......... .......... .......... .......... ..........
A/whooper swan/Mongolia/8/2009    .......... .......... .......... .......... .......... ..........
A/whooper swan/Mongolia/2/2009    .......... .......... .......... .......... .......... ..........

                                          70         80         90        100        110        120
                                  ....|....| ....|....| ....|....| ....|....| ....|....| ....|....|
A/Common Buzzard/BG/38WB/2010     LLGNPLCDEF INVPEWSYIV EKANPANDLC YPGNFNDYEE LKHLLSRINH FEKIQIIPKD
A/great crested-grebe/Qinghai/    .......... .......... .......... .......... .......... ..........
A/grebe/Tyva/3/2009               .......... .......... .......... .......... .......... ..........
A/whooper swan/Mongolia/8/2009    .......... .......... .......... .......... .......... ..........
A/whooper swan/Mongolia/2/2009    .......... .......... .......... .......... .......... ..........

                                         130        140        150        160        170        180
                                  ....|....| ....|....| ....|....| ....|....| ....|....| ....|....|
A/Common Buzzard/BG/38WB/2010     SWSDHEASLG VSAACSYQGN SSFFRNVVWL IKKDNAYPTI KKGYNNTNQE DLLVLWGIHH
A/great crested-grebe/Qinghai/    .......... .....P.... .......... .......... .......... ..........
A/grebe/Tyva/3/2009               .......... .......... .......... .......... .......... ..........
A/whooper swan/Mongolia/8/2009    .......... .......... .......... .......... .......... ..........
A/whooper swan/Mongolia/2/2009    .......... .......... .......... .......... .......... ..........

                                         190        200        210        220        230        240
                                  ....|....| ....|....| ....|....| ....|....| ....|....| ....|....|
A/Common Buzzard/BG/38WB/2010     PNDEAEQTRL YQNPITYISI GTSTLNQRLV PKIATRSKIN GQSGRIDFFW TILKPNDAIH
A/great crested-grebe/Qinghai/    .......... ....T..... .......... .......... .......... ..........
A/grebe/Tyva/3/2009               .......... ....T..... .......... .......... .......... ..........
A/whooper swan/Mongolia/8/2009    .......... ....T..... .......... .......... .......... ..........
A/whooper swan/Mongolia/2/2009    .......... ....T..... .......... .......... .......... ..........

                                         250        260        270        280        290        300
                                  ....|....| ....|....| ....|....| ....|....| ....|....| ....|....|
A/Common Buzzard/BG/38WB/2010     FESNGNFIAP EYAYKIVKKG DSTIMKSEVE YGNCNTRCQT PIGAINSSMP FHNIHPLTIG
A/great crested-grebe/Qinghai/    .......... .......... .......... .......... .......... ..........
A/grebe/Tyva/3/2009               .......... .......... .......... .......... .......... ..........
A/whooper swan/Mongolia/8/2009    .......... .......... .......... .......... .......... ..........
A/whooper swan/Mongolia/2/2009    .......... .......... .......... .......... .......... ..........

                                         310        320        330        340        350        360
                                  ....|....| ....|....| ....|....| ....|....| ....|....| ....|....|
A/Common Buzzard/BG/38WB/2010     ECPKYVKSNK LVLATGLRNS PQRERRRKRG LFGAIAGFIE GGWQGMVDGW YGYHHSNEQG
A/great crested-grebe/Qinghai/    .......... .......... .......... .......... .......... ..........
A/grebe/Tyva/3/2009               .......... .......... .......... .......... .......... ..F.......
A/whooper swan/Mongolia/8/2009    .......... .......... .......... .......... .......... ..........
A/whooper swan/Mongolia/2/2009    .......... .......... .......... .......... .......... ..........

                                         370        380        390        400        410        420
                                  ....|....| ....|....| ....|....| ....|....| ....|....| ....|....|
A/Common Buzzard/BG/38WB/2010     SGYAADKEST QKAIDGVTNK VNSIIDKMNT QFEAVGREFN NLERRIENLN KKMEDGFLDV
A/great crested-grebe/Qinghai/    .......... .......... .......... .......... .......... ..........
A/grebe/Tyva/3/2009               .......... .......... .......... .......... .......... ..........
A/whooper swan/Mongolia/8/2009    .......... .......... .......... .......... .......... ..........
A/whooper swan/Mongolia/2/2009    .......... .......... .......... .......... .......... ..........

                                         430        440        450        460        470        480
                                  ....|....| ....|....| ....|....| ....|....| ....|....| ....|....|
A/Common Buzzard/BG/38WB/2010     WTYNAELLVL MENERTLDFH DSNVKNLYDK VRLQLKDNAK ELGNGCFEFY HKCNNECMES
A/great crested-grebe/Qinghai/    .......... .......... .......... .......... .......... ..........
A/grebe/Tyva/3/2009               .......... .......... .......... .......... .......... ..........
A/whooper swan/Mongolia/8/2009    .......... .......... .......... .......... .......... ..........
A/whooper swan/Mongolia/2/2009    .......... .......... .......... .......... .......... ..........

                                         490        500        510        520        530        540
                                  ....|....| ....|....| ....|....| ....|....| ....|....| ....|....|
A/Common Buzzard/BG/38WB/2010     VRNGTYDYPQ YSEEARLKRE EISGVKLESI GIYQILSIYS TVASSLVLAI MMAGLSLWMC
A/great crested-grebe/Qinghai/    .......... .......... .......... .......... .......... ..........
A/grebe/Tyva/3/2009               .......... .......... .......... .......... .......... ..........
A/whooper swan/Mongolia/8/2009    .......... .......... .......... .......... .......... ..........
A/whooper swan/Mongolia/2/2009    .......... .......... .......... .......... .......... ..........

                                         550        560
                                  ....|....| ....|....| ....|.
A/Common Buzzard/BG/38WB/2010     SNGSLQCRIC I*VCEFKL*L KTPLFL
A/great crested-grebe/Qinghai/    .......... .*....R.*. ......
A/grebe/Tyva/3/2009               .......... .*....R.*. ..
A/whooper swan/Mongolia/8/2009    .......... .*....R.*
A/whooper swan/Mongolia/2/2009    .......... .*....R.*
```

Technical Appendix Figure. Comparison of deduced amino acid sequences of the hemagglutinin protein of A/common buzzard/Bulgaria/38WB/2010 and closely related sequences of 4 other viruses

in the National Center for Biotechnology Information database (A/great crested grebe/Qinghai/1/2009, A/grebe/Tyva/3/2009, A/whooper swan/Mongolia/8/2009, and A/whooper swan/Mongolia/2/2009). Identical amino acids are represented by dots. Potential glycosylation sites are shown as underlined 3-letter sequences. The conserved residues of the receptor-binding site are shaded, and the cleavage polybasic motif is boxed.

Reference

1. Shaw M, Cooper L, Xu X, Thompson W, Krauss S, Guan Y, et al. Molecular changes associated with the transmission of avian influenza A H5N1 and H9N2 viruses to humans. J Med Virol. 2002;66:107–14. PubMed http://dx.doi.org/10.1002/jmv.2118
